# Preoperative prediction of microvascular invasion risk in hepatocellular carcinoma with MRI: peritumoral versus tumor region

**DOI:** 10.1186/s13244-024-01760-2

**Published:** 2024-08-01

**Authors:** Guangya Wei, Guoxu Fang, Pengfei Guo, Peng Fang, Tongming Wang, Kecan Lin, Jingfeng Liu

**Affiliations:** 1 Clinical Oncology School of Fujian Medical University, Fujian Cancer Hospital, Fuzhou, 350014 China; 2 Department of Hepatopancreatobiliary Surgery, Mengchao Hepatobiliary Hospital of Fujian Medical University, Fuzhou, China; 3 Southeast Big Data Institute of Hepatobiliary Health, Mengchao Hepatobiliary Hospital of Fujian Medical University, Fuzhou, China; 4 Department of Radiology, Henan Province Hospital of TCM, Zhengzhou, China; 5 Department of Hepatopancreatobiliary Surgery, First Affiliated Hospital of Fujian Medical University, Fuzhou, China; 6 Department of Hepatopancreatobiliary Surgery, Clinical Oncology School of Fujian Medical University, Fujian Cancer Hospital, Fujian Key Laboratory of Advanced Technology for Cancer Screening and Early Diagnosis, Fuzhou, China

**Keywords:** Hepatocellular carcinoma, Microvascular invasion, Deep learning, Dynamic contrast-enhanced magnetic resonance imaging, Peritumoral region

## Abstract

**Objectives:**

To explore the predictive performance of tumor and multiple peritumoral regions on dynamic contrast-enhanced magnetic resonance imaging (MRI), to identify optimal regions of interest for developing a preoperative predictive model for the grade of microvascular invasion (MVI).

**Methods:**

A total of 147 patients who were surgically diagnosed with hepatocellular carcinoma, and had a maximum tumor diameter ≤ 5 cm were recruited and subsequently divided into a training set (*n* = 117) and a testing set (*n* = 30) based on the date of surgery. We utilized a pre-trained AlexNet to extract deep learning features from seven different regions of the maximum transverse cross-section of tumors in various MRI sequence images. Subsequently, an extreme gradient boosting (XGBoost) classifier was employed to construct the MVI grade prediction model, with evaluation based on the area under the curve (AUC).

**Results:**

The XGBoost classifier trained with data from the 20-mm peritumoral region showed superior AUC compared to the tumor region alone. AUC values consistently increased when utilizing data from 5-mm, 10-mm, and 20-mm peritumoral regions. Combining arterial and delayed-phase data yielded the highest predictive performance, with micro- and macro-average AUCs of 0.78 and 0.74, respectively. Integration of clinical data further improved AUCs values to 0.83 and 0.80.

**Conclusion:**

Compared with those of the tumor region, the deep learning features of the peritumoral region provide more important information for predicting the grade of MVI. Combining the tumor region and the 20-mm peritumoral region resulted in a relatively ideal and accurate region within which the grade of MVI can be predicted.

**Clinical relevance statement:**

The 20-mm peritumoral region holds more significance than the tumor region in predicting MVI grade. Deep learning features can indirectly predict MVI by extracting information from the tumor region and directly capturing MVI information from the peritumoral region.

**Key Points:**

- We investigated tumor and different peritumoral regions, as well as their fusion.
- MVI predominantly occurs in the peritumoral region, a superior predictor compared to the tumor region.
- The peritumoral 20 mm region is reasonable for accurately predicting the three-grade MVI.

**Graphical Abstract:**

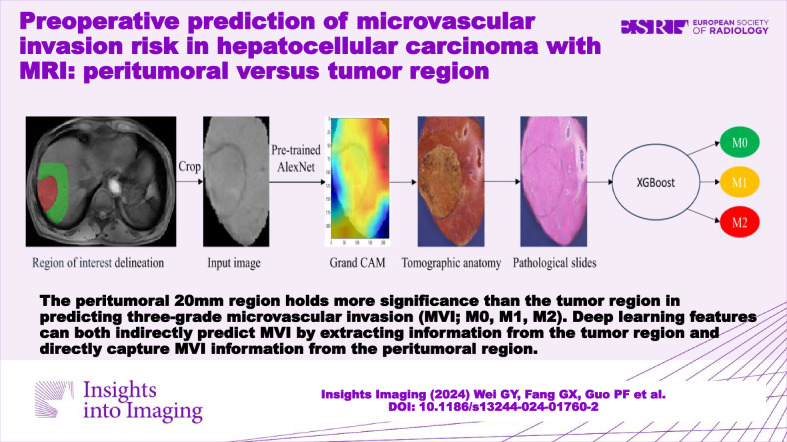

## Introduction

Hepatocellular carcinoma (HCC) is a prevalent malignant tumor, ranking third in cancer-related mortality worldwide [1]. The prognosis for HCC patients remains unfavorable mainly due to the high recurrence rate of the tumor [2]. Microvascular invasion (MVI) refers to the microscopic infiltration of tumor cells into small hepatic vessels, including the microvessels of the portal vein or hepatic artery, and small lymphatic vessels [3], which can only be observed under a microscope and are primarily located in peritumoral or nontumor liver tissue. MVI is considered a crucial pathological factor, as it significantly contributes to increased recurrence rates and reduced survival in patients with liver cancer [4, 5], and has a reported incidence ranging from 15% to 57.1% in HCC patients [6]. The risk of MVI in HCC can be classified into one of three grades [7]: M0 refers to the absence of MVI, M1 (the low-risk category) indicates ≤ 5 sites of MVI, all in the peritumoral hepatic tissue (≤ 1 cm), and M2 (the high-risk category) refers to > 5 sites of MVI or MVI occurring in the distant peritumoral hepatic tissue (> 1 cm). Generally, the overall survival (OS) and recurrence-free survival (RFS) rates in the M1 and M0 groups are higher than those in the M2 group. Compared with nonanatomical liver resection, anatomical liver resection within the M2 group is associated with better OS and RFS rates [8]. Expanding the resection range during hepatectomy can significantly increase the survival rate by eradicating micrometastases [9]. Patients in the M2 group demonstrate increased tumor invasiveness and a greater risk of poor prognosis, and thus surgical resection may not be the optimal choice for these patients; instead, a comprehensive approach involving alternative treatment methods, including liver transplantation, radiotherapy, chemotherapy, targeted therapy, and immunotherapy, should be considered. Therefore, assessing the extent of micrometastasis and distinguishing between M1 and M2 populations before treatment would be beneficial for guiding personalized therapy and improving patient prognosis. However, MVI can only be confirmed through time-consuming postoperative pathological examination. Biopsies are limited by their lack of sensitivity in assessing MVI, tumor heterogeneity, sampling errors, and potential complications. Certain radiological features, such as tumor size [10–12], irregular tumor margins [13–16], the absence of or an incomplete radiological capsule [11, 17], and peritumoral enhancement observed on dynamic contrast-enhanced magnetic resonance imaging (DCE-MRI) [18], regarded as predictors of MVI, and classifications based on “semantic” features are often more easily interpreted and accepted by radiologists. However, this approach is subjective and has poor repeatability. Recently, substantial progress has been made in the field of medical image analysis by utilizing data mining technology, leading to a relatively new field known as radiomics [19]. The gradual application of radiomics in the preoperative prediction of MVI in HCC patients has shown promising results [14, 20, 21]; however, some issues remain. First, most models for predicting MVI primarily focus on tumor-related features and fail to incorporate information from the peritumoral region, where MVI typically manifests and which may provide crucial information [22, 23]. Several studies have also explored the use of tumor and peritumoral radiomic signals for the prediction of preoperative MVI [17, 20, 24, 25], However, the absence of a clear contrast among the various peritumoral regions and the tumor region raises questions about the significance of peritumoral information at different ranges compared to tumor information alone in predicting MVI. Moreover, most studies tend to focus on the qualitative assessment of MVI by determining its presence or absence, and there is a notable scarcity of research exploring the severity of MVI in cases where it is present [26, 27]. AlexNet has revolutionized deep learning and computer vision with its groundbreaking convolutional neural network (CNN)-based architecture. The deep learning features, extracted from a variety of pre-trained CNNs, can be used in the subsequently screened for relevancy and construct a predictive model. This approach employs machine learning techniques to preoperatively predict the MVI status [23]. However, little research has been conducted on the relationship between deep learning features from the different peritumoral regions of HCCs ≤ 5 cm and the three risk grades (M0, M1, M2) of MVI.

Therefore, we applied a pre-trained AlexNet to extract deep learning features from different regions including the tumor and peritumoral regions (5 mm, 10 mm, and 20 mm), and combinations of the tumor and the different peritumoral regions. Our aim was to identify an optimal peritumoral range for the preoperative prediction of the risk grade MVI and to demonstrate that radiomics can potentially not only indirectly predict pathology but also directly capture pathological changes in HCC.

## Materials and methods

### Study design and patient population characteristics

**Fig. 1:**
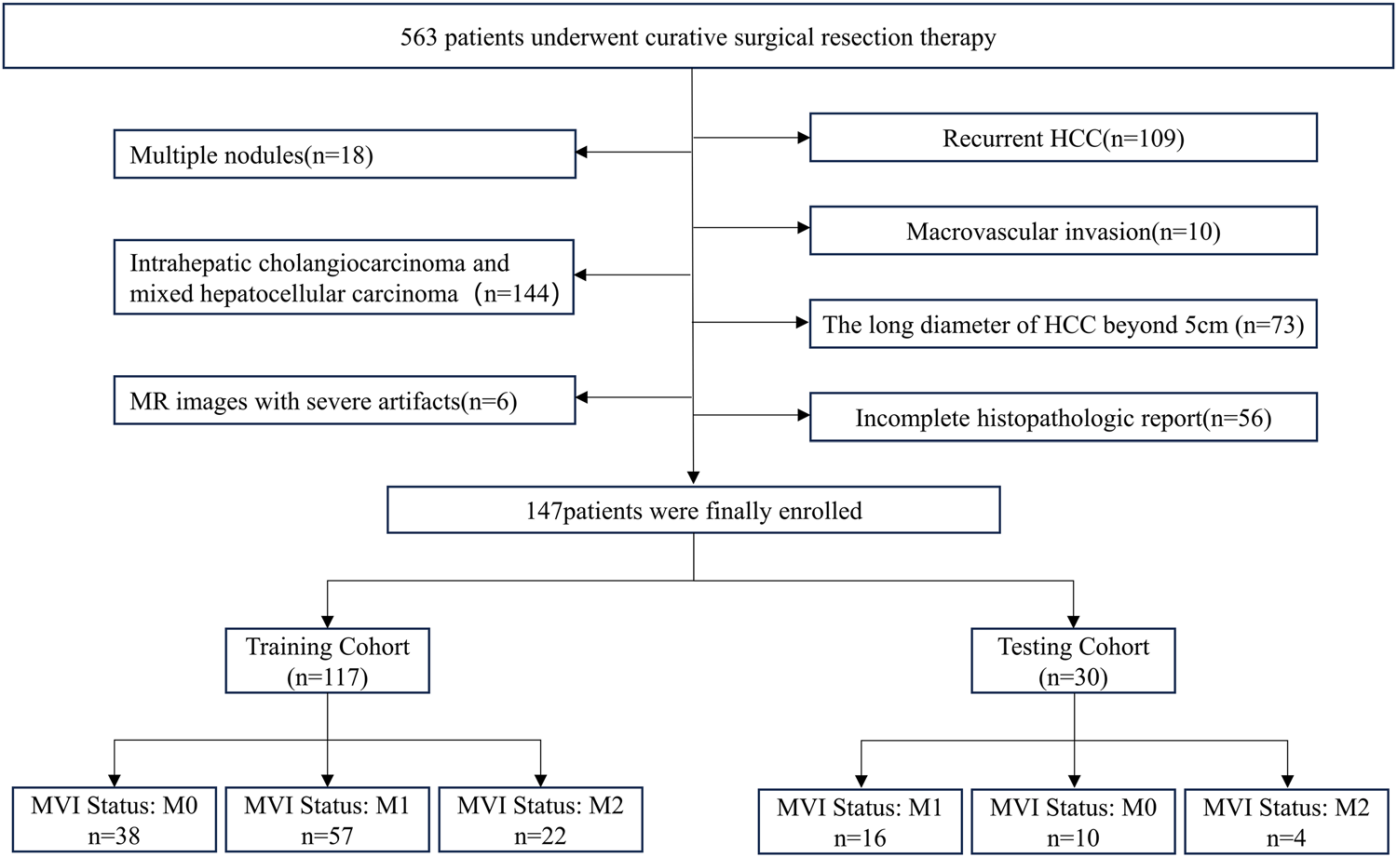
Flow chart of patients’ recruitment for the study. MVI, microvascular invasion. M0, no MVI detected; M1 (low-hazard category), ≤ 5 MVIs all occurring in the peritumoral liver tissue (≤ 1 cm); M2 (high-hazard category), > 5 MVIs or MVI occurring in the distant peritumoral liver tissue (> 1 cm)

This retrospective study (IRB 2021-048-01) at Mengchao Hepatobiliary Hospital, Fujian Medical University, between April 2015 and January 2022, included 563 patients who underwent surgical resection for HCC, diagnosed per World Health Organization criteria. Ethical approval was obtained, and written consent was waived. Inclusion criteria: single HCC lesion ≤ 5 cm post-surgery with complete MVI info and preoperative DCE-MRI within 15 days. Exclusion criteria: (1) recurrent or multifocal HCC, or combined intrahepatic cholangiocarcinoma; (2) antitumor treatment before the enhanced MR scan; (3) radiologically evident invasion of major vessels; and (4) poor imaging data unsuitable for delineating regions of interest (ROIs). After excluding 416 patients, the final analysis comprised 147 patients (28 females, 119 males; mean age 55.71 ± 11.67 years), split into training (*n* = 117) and testing sets (*n* = 30) based on surgery dates (Fig. 1).

### Clinicopathologic factors and MVI

Demographic data (including sex and age), blood biochemistry results (such as neutrophil count (NC) and alpha-fetoprotein (AFP) level, and pathological findings (for example, maximum tumor diameter (MTD)), were obtained from the electronic health records system; Table 1 shows all clinical parameters obtained for the patients. Missing values were imputed by using the mean for continuous variables and the median for categorical variables. MVI in HCC was categorized as M0, M1, or M2, following a standardized pathological framework [7].

**Table 1:** Clinicopathologic characteristics of the patients

Variables	Training cohort (*n* = 117)	Validation cohort (*n* = 30)	*p*-value
Female	27 (23.08%)	1 (3.33%)	4.21
Male	90 (76.92%)	29 (96.67%)	3.66
Age (years)	55.08 ± 11.98	58.20 (9.97)	0.51
BMI (kg/m^2^)	23.50 ± 3.07	23.57 (2.85)	0.98
TMD (cm)	2.82 ± 1.00	3.32 ± 0.99	0.72
γ-GT (μmol/L)	59.71 ± 62.57	60.07 ± 55.83	0.97
IBIL (μmol/L)	12.71 ± 5.97	10.95 ± 4.32	0.58
ALP (U/L)	90.58 ± 31.52	87.47 ± 25.83	0.68
AST (U/L)	33.51 ± 15.54	33.87 ± 15.56	0.95
ALB (g/L)	39.89 ± 4.67	40.97 ± 4.29	0.72
AST /ALT	1.14 ± 0.45	1.05 ± 0.37	0.92
DBIL (μmol/L)	3.67 ± 2.49	2.53 ± 1.06	0.55
ALT (U/L)	34.21 ± 21.33	35.83 ± 22.10	0.81
MONO (*109/L)	0.35 ± 0.14	0.37 ± 0.11	0.97
EOS	2.82 ± 2.92	2.94 ± 2.50	0.96
WBC (*10^9^/L)	5.19 ± 1.76	5.97 ± 1.69	0.67
HGB (g/L)	140.79 ± 17.96	143.23 ± 14.26	0.67
RBC (*10^12^/L)	4.58 ± 0.64	4.61 ± 0.60	0.98
NC	3.00 ± 1.30	3.66 ± 1.56	0.7
APTT (seconds)	36.81 ± 3.96	36.03 ± 3.63	0.78
PT (seconds)	13.48 ± 1.16	13.12 ± 1.11	0.81
Glu (mmol/L)	5.63 ± 0.37	6.00 ± 1.70	0.83
TG (mmol/L)	1.26 ± 0.80	1.34 ± 0.78	0.95
TC (mmol/L)	4.29 ± 1.07	4.56 ± 0.68	0.84
HDL (mmol/L)	1.23 ± 0.34	1.12 ± 0.29	0.89
LDL (mmol/L)	2.31 ± 0.68	3.06 ± 0.73	0.53
CK (mmol/L)	93.74 ± 46.01	141.1 ± 246.47	0.006
AFP (ng/mL)	412.79 ± 981.76	341.23 ± 691.02	0.08
PaO2	94.24 ± 12.08	94.07 ± 11.03	0.97
PCO2	42.81 ± 3.71	44.60 ± 3.03	0.49

*BMI* body mass index, *MTD* maximum tumor diameter, *γ-GT* γ-glutamyl transpeptidase, *IBIL* indirect bilirubin, *ALP* alkaline phosphatase, *ALT* alanine aminotransferase, *ALB* albumin, *AST* aspartate transaminase, *DBIL* direct bilirubin, *MONO* monocyte count, *EOS* eosinophil, *WBC* white blood cell, *HGB* hemoglobin, *RBC* red blood cell, *NC* neutrophil count, *APTT* activated partial thromboplastin time, *PT* prothrombin time, *Glu* glucose, *TG* triglyceride, *TC* total cholesterol, *HDL* high-density lipoprotein, *LDL* low-density lipoprotein, *CK* creatine kinase, *AFP* alpha-fetoprotein, *PaO2* Oxygen partial pressure, *PCO2* partial pressure of carbon dioxide

### DCE-MRI acquisition

MRI was conducted on a 3.0 T scanner (Magnetom Verio, Siemens Healthineers). Dynamic T1-weighted images were acquired using a three-dimensional volume interpolated breath-hold examination fat suppression sequence (t1_vibe_fs_tra_caipi3_bh_pre, FS: 3, TR: 4.5 ms, TE: 2 ms, matrix: 256 × 256, slice thickness: 3 mm). Gadopentetic acid was administered intravenously at a rate of 2 mL/s at a dose of 0.1 mmol/kg. Four routine abdominal DCE-MRI sequences were employed, consisting of the precontrast phase, arterial phase (20–30 s), portal venous phase (approximately 60 s), and delayed-phase sequences (3 min).

### Radiomics analysis

**Fig. 2:**
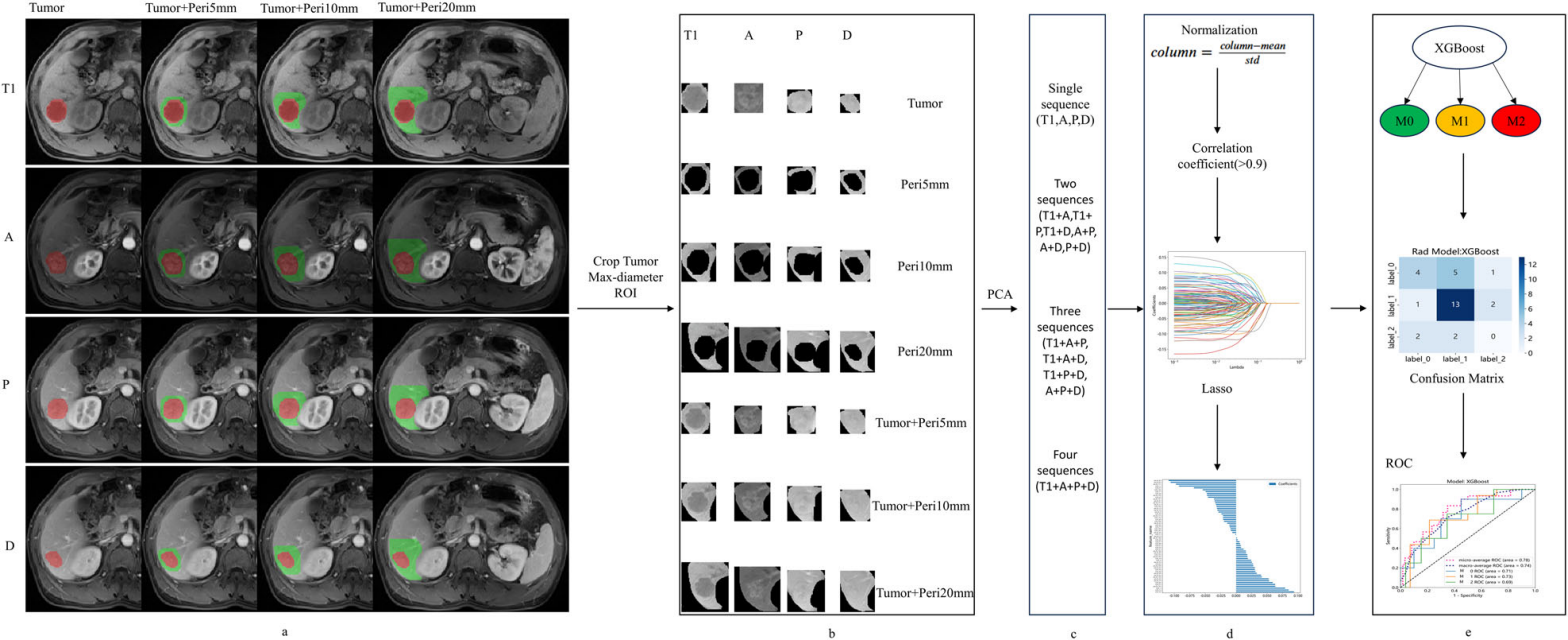
Flow chart of radiomics analysis. **a** Image segmentation: Red represents the tumor area, while green represents the peritumoral area. **b** Deep learning feature extraction (AlexNet pre-trained on MedicalNet). **c** ROI feature fusion d feature selection. **d** Feature selection. **e** Medel construction and evaluation. Tumor, tumor region, Peri5, peritumoral region 5 mm; Peri10, peritumoral region 10 mm; Peri20, peritumoral region 20 mm; ROI, region of interest; A, arterial phase; P, portal venous phase; ROC, receiver operating characteristic curve; AUC, area under the curve

The radiomics workflow involved five steps, including manual tumor segmentation, feature extraction and selection, fusion of diverse sequences and ROIs, and model development and assessment (Fig. 2).

### Data standardization and ROI delineation

Image preprocessing involved the following steps: (1) application of the N4 bias field correction algorithm to all MR imaging data for normalizing the gray level, and (2) nearest-neighbor interpolation for resampling the images to a voxel size of 1 × 1 × 1 mm³. In the process of image segmentation, a radiologist (P.F.) with 10 years of professional experience manually delineated the complete tumor contour layer by layer using ITK-SNAP (version 3.8) across the precontrast phase, arterial phase, portal venous phase, and delayed phase images. The delineated tumor boundaries were independently verified by another radiologist (T.M.W.) with 15 years of professional experience. In cases where there were inconsistent opinions, the two experts collaborated to reach a consensus. The resulting tumor masks were then expanded along the tumor border by 5 mm, 10 mm, and 20 mm, taking care to avoid or manually remove nonliver regions (Fig. 2a). Our study utilized 2D ROIs by precisely selecting the cross-section that portrayed the tumor’s maximum transverse diameter. This specific section typically indicates the region with the most rapid tumor growth and greatest invasiveness, rendering it the most indicative of the tumor’s characteristics. Subsequently, the images were cropped to the masks of the tumor’s maximum transverse diameter and surrounding peritumoral regions (Fig. 2b).

### Radiomics feature extraction

The cropped images were imported into the version of the AlexNet model pre-trained on ImageNet (https://www.image-net.org). The feature extraction process focused on classifier.6 in the AlexNet model (Fig. 2b), resulting in the extraction of 999 deep learning features. Subsequently, these features were dimensionally reduced to 147 using principal component analysis. Finally, separate and fused modeling was performed using these 147 features (Fig. 2c).

### Fusion of multiple sequences and ROIs

The concept of “fusion” of different sequences involves integrating radiomic features extracted from various MRI sequences. We analyzed four MRI sequences, namely precontrast phase (T1), arterial phase (A), portal venous phase (P), and delayed phase (D) sequences. Each sequence encompassed seven distinct ROIs: the tumor region (Tumor), the 5 mm (Peri5 mm), 10 mm (Peri10 mm), and 20 mm peritumoral regions (Peri20 mm), and the combinations of the tumor region with the three peritumoral regions (Tumor + Peri5 mm, Tumor + Peri10 mm, and Tumor + Peri20 mm) (Fig. 2b).

We also investigated the effects of fusing the sequences two (T1 + A; T1 + P; T1 + D; A + P; A + D; P + D) and three at a time (T1 + A + P; T1 + A + D; T1 + P + D; A + P + D) and of fusing all four at once (T1 + A + P + D) (Fig. 2c).

### Radiomics model development and validation

Initially, we created a training set and a testing set according to the chronological order of the patient’s surgical procedures. we performed *z* score normalization to normalize the imaging features; for each feature, we subtracted the average value and divided the difference by the standard deviation. Next, we evaluated the Pearson correlation coefficients of all the features. For feature pairs exhibiting a correlation coefficient higher than 0.90, we randomly removed one feature. The remaining features were then subjected to feature selection using the least absolute shrinkage and selection operator (LASSO) and ranked based on their importance in predicting the results. Features with higher coefficients according to LASSO regression were finally used as the training data (Fig. 2d). After performing 10-fold cross-validation, a stable and robust model was obtained. Finally, we evaluated the extreme gradient boosting (XGBoost) radiomics model using 10-fold cross-validation in the testing set and assessed the prediction performance of the three-grade MVI classifier using the area under the receiver operating characteristic (ROC) curve (AUC) (Fig. 2e).

### Statistical analysis

Continuous variables are typically represented as the mean ± standard deviation, and comparisons among groups were conducted using the *Z* test. Categorical variables are presented as numbers with corresponding percentages, and significant differences between the two groups were evaluated using the chi-square test. We assessed the predictive performance of the radiomics and clinicopathological features by employing AUC. The statistical analyses were conducted using Python (Anaconda3.exe). A statistically significant difference was defined if the two-sided *p*-value was < 0.05.

## Results

### Performance of deep learning features from a single sequence

**Fig. 3:**
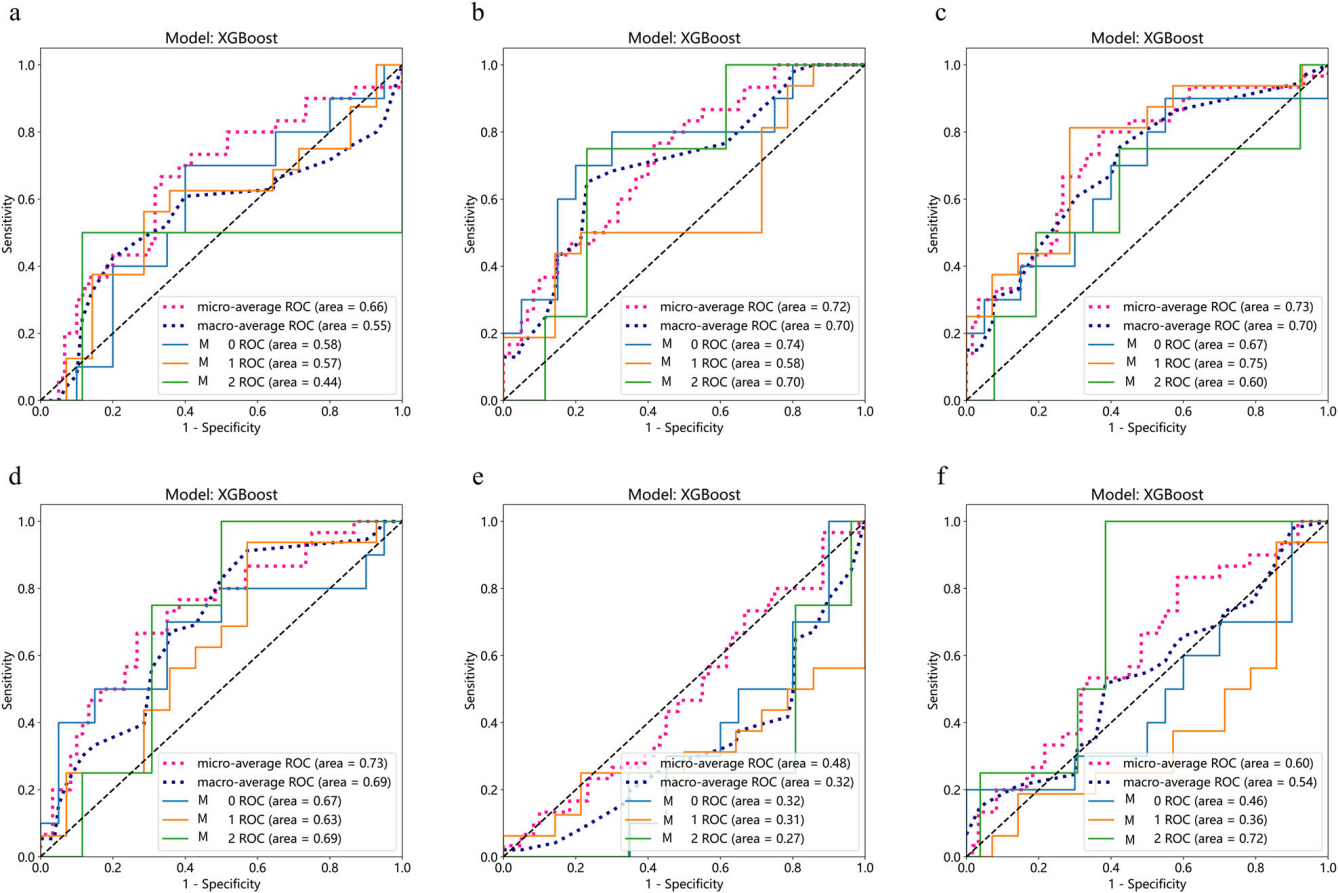
Receiver operating characteristic curves (ROC) of XGBoost model for predicting three-grade MVI in the testing cohort. **a** ROC of the 5 mm peritumoral region in the arterial phase. **b** ROC of the 10 mm peritumoral region in the arterial phase. **c** ROC of the 20 mm peritumoral region in the arterial phase. **d** ROC of the tumor region in the arterial phase. **e** ROC of the tumor region in the portal venous phase. **f** ROC of the 20 mm peritumoral region in the portal venous phase

Table 2 and Fig. 3 present the AUC for each ROI in the single sequence analysis. Notably, the Peri5 mm, Peri10 mm, and Peri20 mm XGBoost classifiers constructed from the arterial-phase data yielded continuous increases in the AUC; specifically, the Peri20 mm model achieved greater predictive performance than the model constructed from the data from the tumor region alone. Furthermore, among models constructed from the portal venous phase data, the predictive performance was better for the Peri20 mm model than for the tumor region alone-based model.

**Table 2:** Results of single sequence based on difference ROI for predicting three-grade MVI (M0, M1, M2) in the testing cohort

Sequence (phase)	ROI	micro-average ROC_AUC	macro-average ROC_AUC
A	Peri5 mm	0.66	0.55
A	Peri10 mm	0.72	0.70
A	Peri20 mm	0.73	0.70
A	Tumor	0.73	0.69
P	Tumor	0.48	0.32
P	Peri20 mm	0.60	0.54

*ROI* region of interest, *A* arterial phase, *P* portal venous phase, *ROC* receiver operating characteristic curve, *AUC* area under the curve, *Tumor* tumor region, *Peri5* *mm* peritumoral 5 mm region, *Peri10* *mm* peritumoral 10 mm region, *Peri20* *mm* peritumoral 20 mm region

### Performance of deep learning features via the fusion of multiple sequences

Table 3 presents the AUC of each ROI in the sequence fusion.

**Table 3:** Results of sequence fusion based on difference ROI for predicting three-grade MVI (M0, M1, M2) in the testing cohort

Sequence-fusion_ROI	micro-average ROC_AUC	macro-average ROC_AUC
P + D_Tumor + Peri5 mm	0.56	0.45
P + D_Tumor + Peri10 mm	0.68	0.65
P + D_Tumor + Peri20 mm	0.74	0.64
A + P_Peri20 mm	0.73	0.67
A + P_Tumor	0.61	0.5
A + D_Peri20 mm	0.69	0.59
A + D_Tumor	0.62	0.56
P + D_Peri20 mm	0.58	0.48
P + D_Tumor	0.56	0.44
A + P + D_Peri5 mm	0.62	0.45
A + P + D_Peri10 mm	0.64	0.57
A + P + D_Peri20 mm	0.67	0.57
A + P + D_Tumor	0.54	0.45
T1 + A + D_Peri20 mm	0.7	0.58
T1 + A + D_Tumor	0.62	0.57
T1 + A + P_Peri20 mm	0.71	0.64
T1 + A + P_Tumor	0.67	0.49
T1 + P + D_Peri20 mm	0.56	0.47
T1 + P + D_Tumor	0.56	0.45
T1 + A + P + D_Tumor + Peri5 mm	0.53	0.39
T1 + A + P + D_Tumor + Peri10 mm	0.6	0.5
T1 + A + P + D_Tumor + Peri20 mm	0.62	0.51
T1 + A + P + D_Peri20 mm	0.63	0.47
T1 + A + P + D_Tumor	0.52	0.46

*T1* precontrast phase, *A* arterial phase, *P* portal venous phase, *D* delayed phase

Among the models constructed from fusing two sequences, the AUCs of the P + D models in predicting MVI grade increased as they incorporated larger peritumoral areas; that is, the model constructed with Tumor+ Peri5mm features yielded the lowest AUC, followed by that constructed from Tumor + Peri10 mm features and that constructed from Tumor + Peri20 mm features. Moreover, for the A + P, A + D, and P + D fusion sequences, the AUC of the Peri20 mm model surpasses that of the tumor region alone (Tumor) model.

Among the models constructed from the fusion of three sequences, the A + P + D models showed increasing AUCs in predicting MVI grade when constructed from Peri5 mm, Peri10 mm, and Peri20 mm features in that order. For fusion models, A + P + D, T1 + A + D, T1 + A + P, and T1 + P + D, the AUC of the Peri20 mm-based model surpassed that of the tumor region alone (Tumor) model.

The AUCs of the models constructed from fusing all four sequences (T1 + A + P + D) in predicting MVI grade increased as the models incorporated features from larger peritumoral areas (i.e., Tumor + Peri5 mm yielded the lowest AUC, followed by Tumor + Peri10 mm and Tumor + Peri20 mm). Moreover, the AUC of the Peri20 mm model surpassed that of the tumor region alone (Tumor) model.

Table 4 illustrates the optimal prediction performance of models constructed from both individual sequences and their fused counterparts. Notably, the models constructed from the fusion of two sequences (T1 + D) exhibited the highest predictive performance when incorporating the tumor and Peri20 mm regions, with a micro-average AUC of 0.78 and a macro-average AUC of 0.74.

**Table 4:** Results of the optimal prediction performance of the single sequence and their fused sequences for predicting Three-grade MVI (M0, M1, M2) in the testing cohort

Sequence-fusion_ROI	Max micro-average ROC_AUC	Max macro-average ROC_AUC
T1_Tumor + Peri20 mm	0.74	0.71
T1+D_Tumor + Peri20 mm	0.78	0.74

### Visualization of deep learning features

**Fig. 4:**
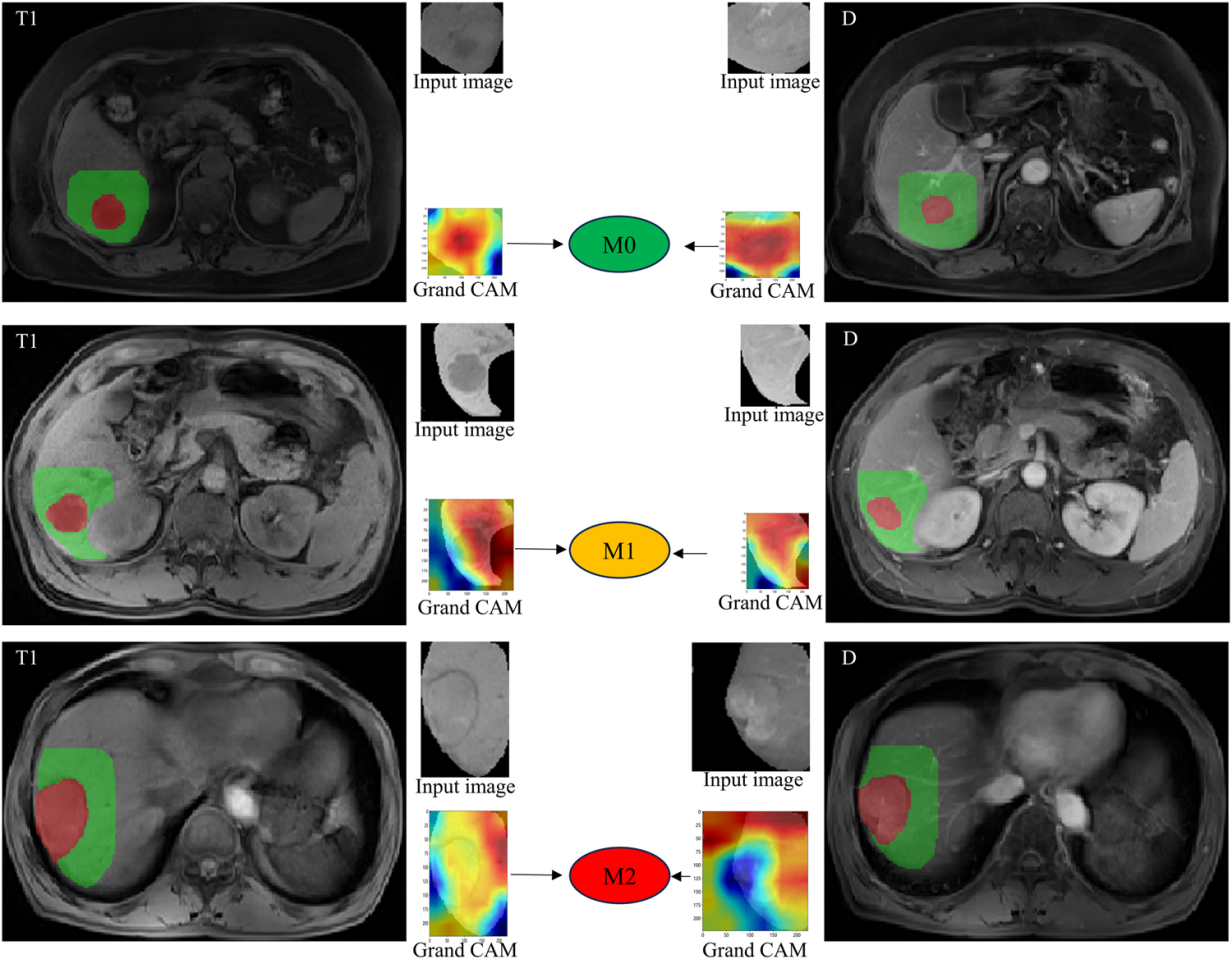
The pre-trained AlexNet model extracts features of Tumor + Peri2  mm region for the precontrast phase (T1) and delayed phase (D) image. Representative examples of attention heatmaps were generated by using the gradient-weighted class activation mapping (Grad-CAM) method Abbreviations: T1, precontrast phase; D, delayed phase; Peri, peritumoral region; Tumor, tumor region

To further elucidate the remarkable and promising findings of this study, we visualized the deep learning features extracted from images labeled as having grades M0, M1, and M2 MVI using the pre-trained AlexNet model (Fig. 4). In the heatmap produced with Grad-CAM, different colors typically indicate different activation strengths: warmer colors signify greater importance for the model’s predictions in the corresponding regions, while cooler tones indicate lower activation strength, suggesting that the model pays relatively less attention to those regions.

### Performance of radiomics and clinicopathologic features

**Fig. 5:**
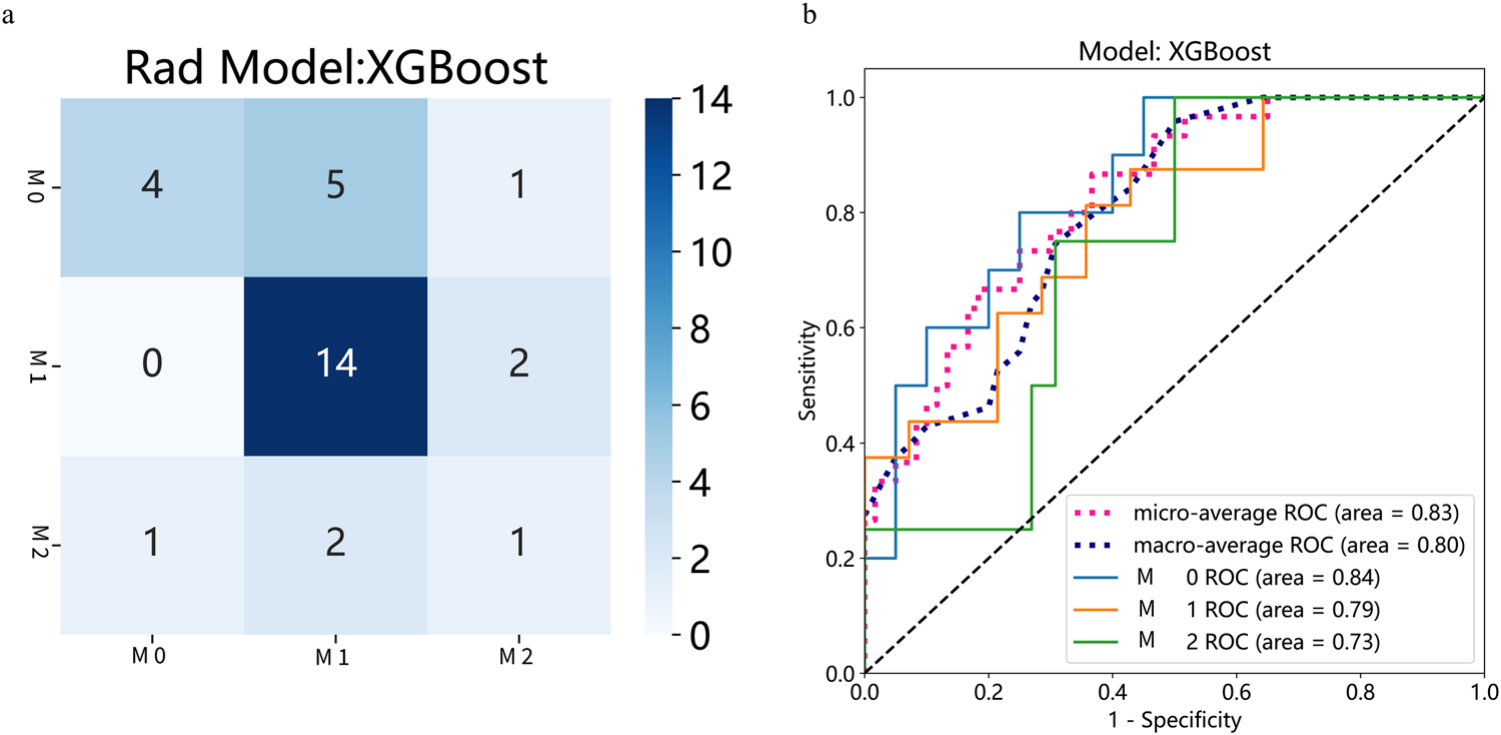
Performance of Clinicopathologic features and deep learning features from the combined region of the tumor and peritumoral 20 mm in two sequences (T1 + D) fusion in the testing cohort. **a** Confusion Matrix of Deep learning and clinicopathologic features predicting three-class MVI. **b** ROC of Clinicopathologic characteristics predicting three-class MVI. Abbreviations: AFP, alpha-fetoprotein; NC, Neutrophil count; MTD, maximum tumor diameter

No statistically significant differences were observed in the baseline characteristics among the subgroups. The overall prevalence of M1 MVI in the cohort was 49.7%, while the overall prevalence of M2 MVI was 17.7%. To further improve the predictive performance of the models, we integrated clinical information with deep learning features. LASSO regression was used to select the final set of deep learning and clinicopathologic features, including the serum AFP level, NC, and TMD. Interestingly, a fusion of the T1 + D_Tumor+Peri20 radiomic features and clinical features achieved a confusion matrix (Fig. 5a) and the highest AUC (0.83, 0.80) across all sequences and regions (Fig. 5b).

## Discussion

This study represents the first attempt, to our knowledge, to employ a pre-trained AlexNet to extract deep learning features from various DCE-MRI sequences depicting tumors and the corresponding 5-, 10-, and 20-mm peritumoral areas and their combinations for predicting the grade of MVI in patients with HCCs ≤ 5 cm. In addition, it includes a comparative analysis of distinct MRI sequences for obtaining the Peri5 mm, Peri10 mm, and Peri20 mm regions. The results demonstrated that as the peritumoral region expanded, the AUC increased; notably, the AUCs of the models constructed from Peri20 mm data region were greater than those of models constructed from data from the tumor region. These results as well as corresponding heatmaps suggest that deep learning features capture more attention-related information about MVI from the peritumoral region. The Peri20 mm region is more important than the tumor region for predicting the grade of MVI, which to our knowledge was first demonstrated here. This research provides further evidence that radiomics-based deep learning features are capable of not only indirectly predicting MVI by extracting relevant information from the tumor region but also directly capturing MVI information from the peritumoral region. There are several possible explanations for this phenomenon: (1) MVI primarily occurs in the peritumoral region, as demonstrated by a study [28] conducted by Kai-Qian Zhou, which revealed among patients with MVI, MVI within 0.5 mm of the tumor margin in 68.0%, within 10 mm in 83.3%, and within 20 mm of the tumor margin in 91.7%. (2) Deep neural networks can unveil hierarchical feature representations, enabling them to derive higher-level features from lower-level features [29]. (3) CNNs can adapt to the intrinsic structure of medical images, making them well-suited for shape recognition [30].

Previous studies on constructing models for predicting MVI have predominantly concentrated on the tumor itself, disregarding investigations of the peritumoral region [31, 32]. While some earlier studies considered peritumoral information, they did not explore margins up to 20 mm, as specified in the diagnostic criteria for MVI [24, 25, 33–36]; moreover, they mainly focused on the qualitative prediction of MVI. Hu, F. et al reported that they explored the 20-mm peritumoral region, mainly utilizing traditional radiomic features without comparing different peritumoral regions [37]. In contrast to these studies, we demonstrated that among the single sequence models, precontrast phase-based models had a superior prediction performance to models separately based on arterial phase, portal venous phase, and delayed phase data. The discrepancy in the results may be attributed to the fact that previous studies utilized traditional radiomics features and logistic regression as the classifier, while our study employed deep learning features and XGBoost as the classifier. Our multisequence fusion analysis revealed that the A + D phase-based models achieved the greatest predictive performance for MVI risk grade when constructed from Tumor + Peri20 mm features. Both the presence or absence of MVI and its severity are important prognostic factors. Accurately identifying the preoperative severity of MVI can help ensure that patients receive more appropriate treatment. A study investigating the three grades of MVI in variously sized peritumoral regions revealed that deep learning features have the ability to indirectly predict clinical pathological indicators and directly observe specific pathological phenomena. Our research, along with related work, differs from previous studies conducted by other teams, as it reveals the potential mapping between deep learning features in the peritumoral regions of HCC and their observable histopathological features. The obtained findings have yielded surprising and enlightening insights.

This retrospective single-center study has several limitations. First, while the dataset was divided into training and testing sets based on surgical time, the TRIPOD statement recommends temporal validation over random grouping [38], To tackle this constraint, the study endeavored to leverage transfer learning, ensemble methods, and 10-fold cross-validation to mitigate overfitting risks and bolster the model’s efficacy on small-sample datasets. Therefore, further prospective multicenter validation in larger cohorts is necessary. Another possible limitation in our radiomic study is the use of two-dimensional (2D) ROIs. Three-dimensional (3D) segmental information provides more informative data, which we will seek to incorporate in future research. Finally, we only conducted a preliminary visual analysis of the features extracted by AlexNet, and our results do not allow us to establish a correlation between the specific location of MVI in tumor tissue and the positions of radiomics features in the peritumoral region. We aim to explore this aspect in future studies.

## Conclusion

In conclusion, our study has yielded promising results in preoperatively predicting the grade of MVI using DCE-MRI of the 20-mm peritumoral region. Importantly, the peritumoral region may provide more direct and important information for predicting the grade of MVI.

## Data Availability

The raw data and corresponding code, if needed, can be obtained from the corresponding authors upon request.

## Abbreviations

AUC: Area under curve
CNNs: Convolutional neural networks
DCE-MRI: Dynamic contrast-enhanced magnetic resonance imaging
HCC: Hepatocellular carcinoma
IRB: Institutional review board
LASSO: Least absolute shrinkage and selection operator
MVI: Microvascular invasion
ROC: Receiver operating characteristic
ROI: Regions of interest
XGBoost: The extreme gradient boosting
